# A bibliometric analysis of worldwide cancer research using machine learning methods

**DOI:** 10.1002/cai2.68

**Published:** 2023-04-11

**Authors:** Lianghong Lin, Likeng Liang, Maojie Wang, Runyue Huang, Mengchun Gong, Guangjun Song, Tianyong Hao

**Affiliations:** ^1^ School of Artificial Intelligence South China Normal University Guangzhou China; ^2^ School of Computer Science South China Normal University Guangzhou China; ^3^ Guangdong Provincial Hospital of Chinese Medicine Guangzhou China; ^4^ Guangdong Provincial Key Laboratory of Clinical Research on Traditional Chinese Medicine Syndrome Guangzhou China; ^5^ State Key Laboratory of Dampness Syndrome of Chinese Medicine The Second Affiliated Hospital of Guangzhou University of Chinese Medicine Guangzhou China; ^6^ Institute of Health Management Southern Medical University Guangzhou China; ^7^ Guangzhou BiaoQi Optoelectronics Co., Ltd. Guangzhou China

**Keywords:** bibliometric analysis, cancer, Latent Dirichlet Allocation, machine learning, research topic, topic evolution

## Abstract

With the progress and development of computer technology, applying machine learning methods to cancer research has become an important research field. To analyze the most recent research status and trends, main research topics, topic evolutions, research collaborations, and potential directions of this research field, this study conducts a bibliometric analysis on 6206 research articles worldwide collected from PubMed between 2011 and 2021 concerning cancer research using machine learning methods. Python is used as a tool for bibliometric analysis, Gephi is used for social network analysis, and the Latent Dirichlet Allocation model is used for topic modeling. The trend analysis of articles not only reflects the innovative research at the intersection of machine learning and cancer but also demonstrates its vigorous development and increasing impacts. In terms of journals, *Nature Communications* is the most influential journal and *Scientific Reports* is the most prolific one. The United States and Harvard University have contributed the most to cancer research using machine learning methods. As for the research topic, “Support Vector Machine,” “classification,” and “deep learning” have been the core focuses of the research field. Findings are helpful for scholars and related practitioners to better understand the development status and trends of cancer research using machine learning methods, as well as to have a deeper understanding of research hotspots.

## Graphical abstract

Applying machine learning methods to cancer research has become an important research field. We summarize the most recent research status and trends, in terms of publication distribution by year, country/region, institution, and authors, as well as author collaboration, research topics, topic evolutions, and potential directions of the field.

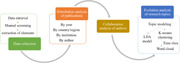

**Abbreviations**
AI: artificial intelligence
CAD: computer aided diagnosis
CNN: convolutional neural network
CT: computed tomography
DL: deep learning
DM: data mining
DT: decision tree
LSTM: long‐short term memory
ML: machine learning
NLP: natural language processing
RF: random forest
RNN: recurrent neural network
SVM: support vector machine
UK: United Kingdom
USA: United States

## 1 INTRODUCTION

The area of artificial intelligence (AI) has a long and intertwined history. As a branch of computer science, AI has the ability to imitate certain thinking processes and intelligent behaviors of humans, for example, “learning” and “problem‐solving” [1]. Machine learning methods have been playing a significantly important role as the core of AI development. Machine learning methods aim to discover features hidden in massive cancer data, which can help make a clinical diagnosis and propose treatment options [2]. They predict and assess cancer conditions, help personalize cancer treatment and help clinicians store medical images, and convert image data into quantitative ones for statistical analysis. In addition, machine learning methods are able to extract essential information from a large amount of clinical data to learn effective patterns for assisting clinical decision‐making [1].

In the past decade, the use of machine learning methods for cancer research has attracted great interest from the scientific community, as can be seen from the annual increase in the number of research papers published [3]. The interest has also raised clinicians’ expectations about the potential influence of machine learning methods on their professional development [4]. Especially in recent years, researchers have been exploring how machine learning methods can be applied to cancer research. For example, Wu et al. [5] combined deep learning and radiomics methods to investigate whether peritumoral had predictive value for tumor data. Their results indicated that peritumors had additional predictive value by leveraging deep learning or radiomics. Jiao et al. [6] used machine learning methods to achieve an automatic assessment of the tumor microenvironment on Giga‐pixel digital histopathology whole‐section images. The method was validated on colon cancer cases from the Cancer Genome Atlas Project. Dominik et al. [7] utilized a calibrated three‐dimensional convolutional neural network (CNN) for risk assessment of multiparametric magnetic resonance imaging (mpMRI) and performed a risk assessment for clinically significant prostate cancer (csPCa) by decision curve analysis (DCA). As a result, the field of cancer research using machine learning methods has developed rapidly and is receiving increasing attention [8]. Therefore, it is of great significance to systematically analyze the existing research publication to understand the recent development status and research hotspots of this research field. At the same time, many journals have published articles analyzing the contributions and influence of academic output in various scientific fields using quantitative approaches, but little is known heretofore in the field of the analysis of worldwide cancer research using machine learning methods. For example, which journals, countries/regions, institutions, and authors contribute more to the field? What are the research themes and their evolutions with time going on?

To fill this gap and help advance the field of cancer research using machine learning methods, this study adopts a bibliometric and topic modeling technique for a systematic review. With the explosive growth of research publication output, the need for a new approach to structural knowledge emerged [9]. Bibliometrics was first proposed and defined as “Mathematical and statistical methods” to clearly indicate “the process of written communication and the developmental characteristics of the discipline expressed by counting and analyzing the number of publications” by Alan Pritchard [10]. Compared with other knowledge synthesis methods, bibliometrics has the capacity for quantitatively analyzing vast amounts of publications on macroscopic and microscopic levels. The use of bibliometrics benefits researchers in multiple aspects and thus has become increasingly popular in various research fields. It has been particularly used in interdisciplinary research, for example, AI on technology hotspot tracking research [11], natural language processing in medical research [12], data mining in medical research [13], and thematic evolution in social media research [14]. In addition, topic modeling is a powerful algorithm in the field of text mining, which could discover the latent thematic structures and relationships among text data [15]. Latent Dirichlet Allocation (LDA) is one of the most commonly used topic modeling algorithms [16].

To that end, this study aims to provide a bibliometric approach to evaluate academic articles about cancer research using machine learning methods published between 2011 and 2021. The goals of this study include: (a) revealing research characteristics and trends of machine learning methods for cancer research in the period of 2011–2021; (b) summarizing contributions on the ground of journals, countries/regions, institutions, and authors; (c) exploring scientific collaborative relations among authors; and (d) identifying main research topics and how they evolve with time through keywords analysis.

## 2 METHODOLOGY

**Figure 1:**
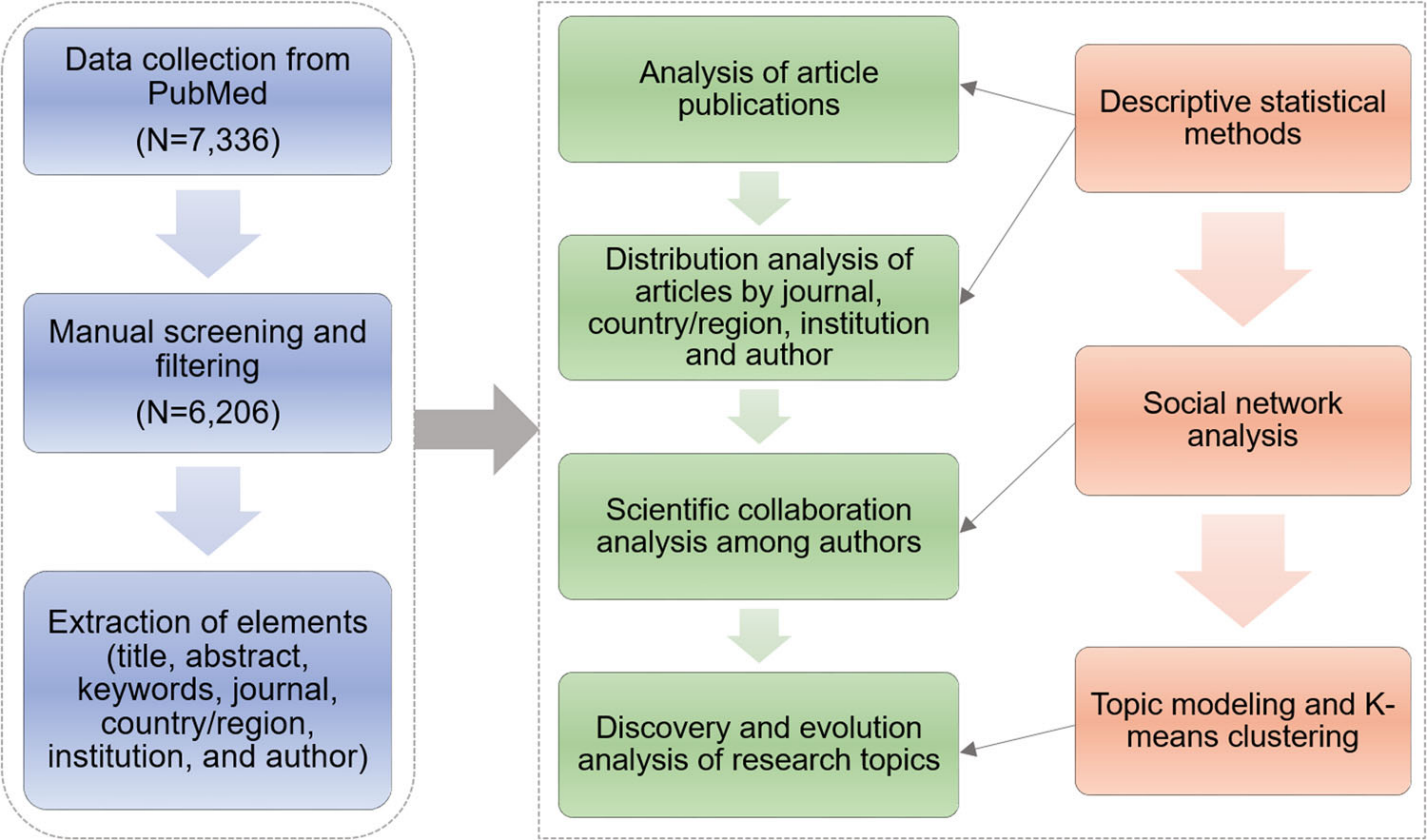
The flowchart of data acquisition and analysis for using machine learning methods for cancer research.

This paper designs a flowchart of data collection and analysis for analyzing articles about using machine learning methods for cancer research. As shown in Figure 1, the overall processing architecture can be categorized into five subprocesses: (a) data retrieval and preprocessing; (b) evolution analysis of article publications; (c) distribution analysis of articles by journal, country/region, institution, and authors; (d) scientific collaboration analysis among authors; and (e) discovery and evolution analysis of research topics. These subprocesses are elaborated in the following subsections sequentially. Considering Python, as a popular programming language, has abundant natural language processing libraries, high data format compatibility, and powerful parallel processing capability, this study mainly uses Python programming to carry out the analysis.

### 2.1 Data retrieval and preprocessing

A bibliometric methodology is applied by collecting relevant publications in the research field from PubMed. PubMed provides a large amount of biomedicine‐related publications, which are closely related to the subject and has been widely applied for bibliometric analysis. Data were retrieved from the PubMed database (https://pubmed.ncbi.nlm.nih.gov) on May 29, 2022.

In the database, Title and MeSH terms are used as two search fields. Combining with the “Title” and “MeSH terms” retrieval manner, a search query is defined as ((“Machine learning”) OR (“Data Mining”) OR (“deep learning”)) AND ((“Neoplasms”) OR (“cancer”) OR (“tumor”)). The search query is applied and is further restricted by the following conditions: (a) Considering the rapid development and change of machine learning technologies, the articles published during the period 2011–2021 are used only to more precisely study the recent development of this field in the last decade; (b) articles of “ARTICLE” type; (c) articles written in English; (d) Journal category is “MEDLINE”; (e) the species of study is “Humans.” According to the above restrictions, a total of 7336 articles with complete bibliographic information and abstracts are retrieved. To ensure the high relevance of the retrieved publications, a specific exclusion strategy is also designed in this study. Records without “Machine Learning” and “Cancer” related terms in the title or abstract are filtered out through a manual screening. The manual screening strategy of the papers is as follows. First, a domain expert reads the abstracts of a certain number of publications, preliminarily screening these publications and sorting out commonly used terms related to “cancer” and “machine learning,” such as “support vector machine,” “convolutional neural network,” and “breast cancer.” Then, these terms are used for initial screening, and records containing these terms in titles or abstracts are included. Finally, titles and abstracts of the publication are read by the expert to exclude records that either did not focus on cancer research or did not involve machine learning techniques. After filtering, 6206 articles are obtained. Raw data are exported in plain text form, in which key elements, for example, title, published year, abstract, and author address, are automatically extracted. The affiliations and countries' information of authors is identified according to author affiliations and addresses. Inconsistent expressions or terms are standardized to be consistent manually. The search strategy with keywords used is presented in Table 1.

**Table 1:** The search strategy with keywords used in retrieving relevant publications from PubMed.

Search scope	Title and MeSH terms
Keyword strategy	Extend keywords related to “Machine Learning” and “Cancer”
Search query	(("Machine learning"[Mesh]) OR ("Data Mining"[Mesh]) OR ("deep learning "[Mesh])) AND (("Neoplasms"[Mesh]) OR("cancer") OR("tumor"))
Query operator	AND
Search criteria	Timespan: 2011–2021
	Species: Humans
	Source: Journal Article
	Journal category: MEDLINE
Retrieved in total	7596 records
After excluding records without abstract	7336 records
After excluding records without “Machine Learning” and “Cancer” related terms in Title OR Abstract	6206 records

### 2.2 Social network analysis

Social network analysis is used to characterize coauthorship and has the characteristics of nondirectional. This study builds collaboration networks in three steps by resorting to Gephi [17, 18]. First, input data are preprocessed to form a node table and a link table. The node table consists of three columns, namely, article id, author name, and the number of articles. In the link table, there are three columns, that is, source and target are used as corresponding author names for recording the collaborations, as well as weight to indicate the number of collaborative articles. Second, based on the node table and link table, a coauthorship network is constructed using the layout of the Fruchterman‐Reingold algorithm. Last, the node size and node color are automatically adjusted based on article count and data group identities by computing the weights.

During the network construction, the Fruchterman‐Reingold Algorithm is a force‐directed layout algorithm whose core idea is to consider a force between any two nodes. In the algorithm, the nodes are represented by steel rings and the edges between nodes are springs. An attractive force is analogous to a spring force and a repulsive force is analogous to an electrical force. The energy of the whole system is minimized by moving nodes and changing the forces between them. The sum of the force vectors determines which direction a node should move. The step width, which is a constant determined how far a node moved in a single step. When the energy of the system is minimized, the nodes stop moving, and the system reaches an equilibrium state [19].

The attractive and repulsive forces are set to $f_{a}$ and $f_{r}$, as Equations (1) and (2), respectively, in the algorithm.

(1) $$f_{a}\left(d\right)=\frac{d^{2}}{k},$$

(2) $$f_{r}\left(d\right)=\frac{{-k}^{2}}{d},$$

*d* is the distance between the two vertices. As for the optimal distance between two vertices, *K* is defined as Equation (3).

(3) $$K=c\times \sqrt{\frac{W\times L}{\text{number of vectices}}}\;,$$

*W* and *L* are the width and length of the frame and *C* is constant.

### 2.3 Topic modeling

To discover major research topics in the collected articles, LDA is applied, which is a topic model that has been widely adopted in many different domains and contexts [20, 21]. The topic modeling analysis is conducted in the following three steps. First, article keywords provided by authors often accurately and comprehensively summarize the main information and key focus of a study and are frequently used to reveal research topics with their frequencies [22]. In addition to the article keywords, Keywords Plus and PubMed MeSH, the keywords in the title and abstract are extracted for deep usage. To perform topic modeling adequately and effectively, keywords are preprocessed according to the following three strategies. (a) Abbreviations are replaced by their full names, depending on the relevant article context (e.g., “CNN” is replaced by “Convolutional neural networks,” “SVM” is replaced by “Support Vector Machine,” and “DL” is replaced by “Deep Learning”). (b) All keywords are uniformly set to lowercase. (c) Some keywords with little contribution to the topic analysis such as “based,” “purpose,” “article,” and “using,” are removed. (d) Keywords that are semantically repeated (e.g., “behavior” and “behaviour”) are merged consistently.

Second, the term frequency‐inverse document frequency (TF‐IDF) method is used for the selection of important keywords. A threshold is set to 0.2 empirically by inspecting ranked terms, that is, only terms with TF‐IDF > 0.2 are included. Third, 16 topics are selected out based on the perplexity criteria [23]. These 16 thematic results are interpreted to assign labels by examining representative terms for each topic and referring to related articles.

In addition, a clustering model is also applied to cluster the articles. The clustering model is the process of dividing samples into multiple classes composed of similar objects [24]. After clustering, it is possible to explore correlations and major differences between different groups [25]. We use the K‐means algorithm, which minimizes the objective function by separating samples into groups with equal variances. The algorithm requires a specified number of clusters, scales well to a large‐scale sample, and has been widely used in a range of application domains [26]. The purpose is to separate data to enable that the data in the same cluster are similar, while the data in different clusters are different [27, 28]. Different from the LDA model, the data used is from author keywords, Keywords Plus, and PubMed MeSH.

Besides, word cloud analysis is performed using the WordArt tool. Word cloud emphasizes keywords with high frequency in articles in a visual way, forming a keyword cloud or “keyword rendering for quickly and visually identify essential information” [29]. In a word cloud, font size indicates the frequency of keyword occurrences. Based on this, the development trend of each keyword over time is developed and visualized [30].

### 2.4 Main indicators

*Publications* are the number of articles produced. Publication quantity is one of the critical indexes to measure scientific research production capacity in bibliometrics [31].

*The impact factor* also as the impact index or impact coefficient refers to the frequency of citations of articles in a journal compared with its article quantity in a specific year or period [32, 33]. It is a valuable indicator to measure the influence of academic journals [34].

*H‐index* is a hybrid quantitative index, originally proposed by Jorge Hirsch, which aims to quantify the research achievements of researchers as individuals [35]. If the published results of a scientist are ranked according to the number of their citation life cycle, the *H*‐index is a maximum value, which refers to the *H* articles cited at least *H* times for each article [36]. The initial application of the *H*‐index is to measure the scientific output of individual scientists [37, 38]. Due to its simplicity and ease of use, *H*‐index has been extended to the evaluation of other fields such as journals, countries, institutions, and disciplines [39].

## 3 THE RESULTS

We report the analysis results including the trends in article publications, top journals, influential countries/regions, institutions, as well as authors, scientific collaborative relations among authors, and major research topics.

### 3.1 Analysis of article publications

**Figure 2:**
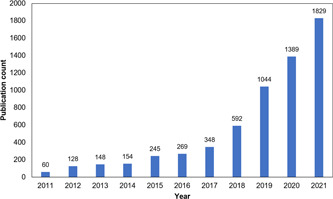
Trend analysis of publication counts.

The evolution of scientific publications over time is an essential aspect to evaluate the development of a field, and a comprehensive statistical analysis of it is of great significance to evaluate the development trend and dynamics of this field [40]. Based on the statistics of publications on cancer research using machine learning methods, a chart is drawn to demonstrate the change in the number of publications, as shown in Figure 2. The number of publications showed a rising trend in general from 2011 to 2021. Meanwhile, the research field has been heating up around the world in the recent 10 years. From 2011 to 2016, the number of publications was relatively low and stable. Since the epidemic of AI in 2016, the number of publications has shown a trend of substantial growth.

**Figure 3:**
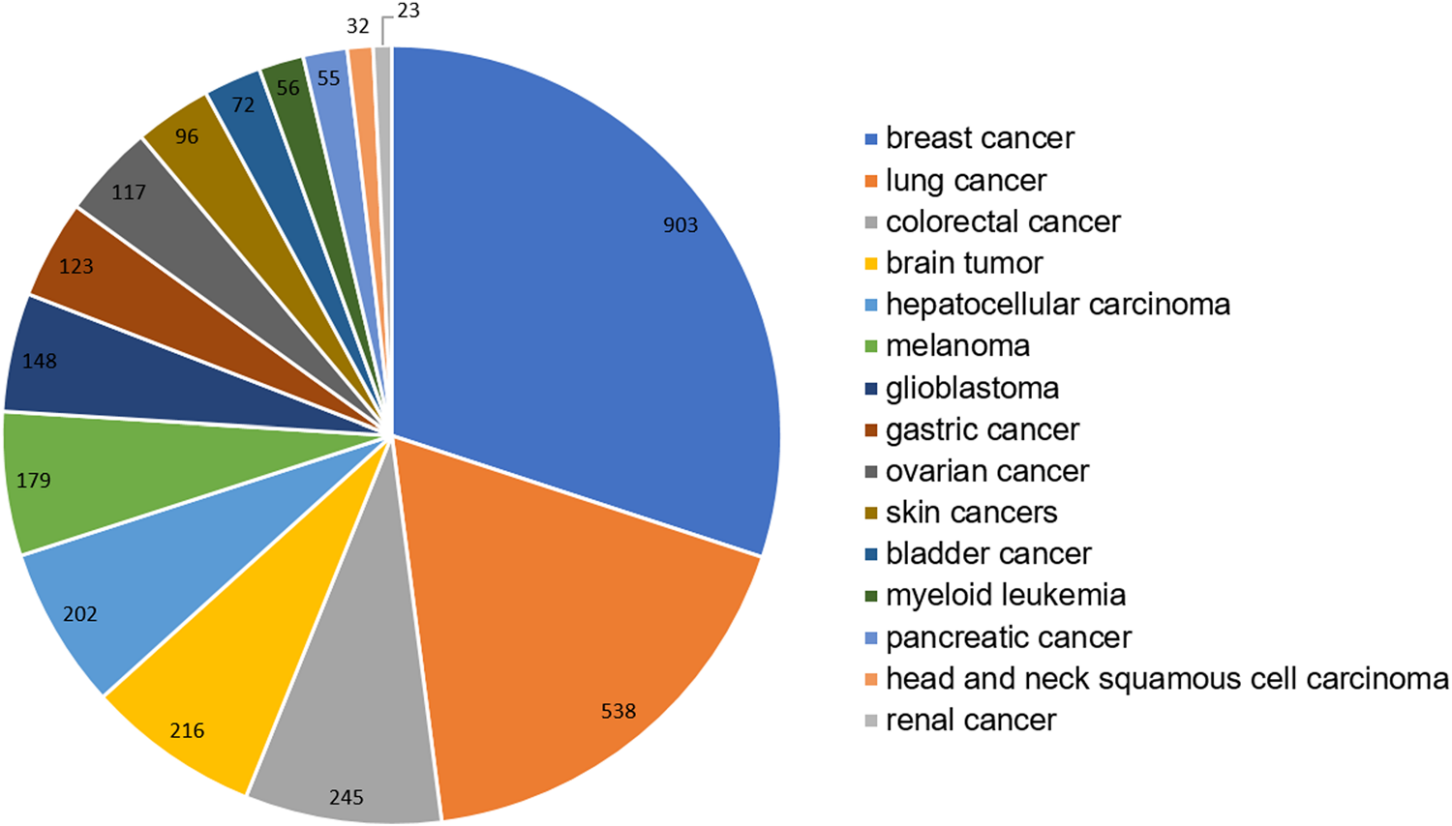
Distribution of cancer types involved in the article publications.

Among the article publications related to cancer research, a variety of cancer types are distributed. The statistics of cancer types studied help understand the current situation of cancer research using machine learning methods. As shown in Figure 3, in this decade, studies on “breast cancer” (903 articles) are the most frequent, followed by studies on “lung cancer” (538 articles).

**Figure 4:**
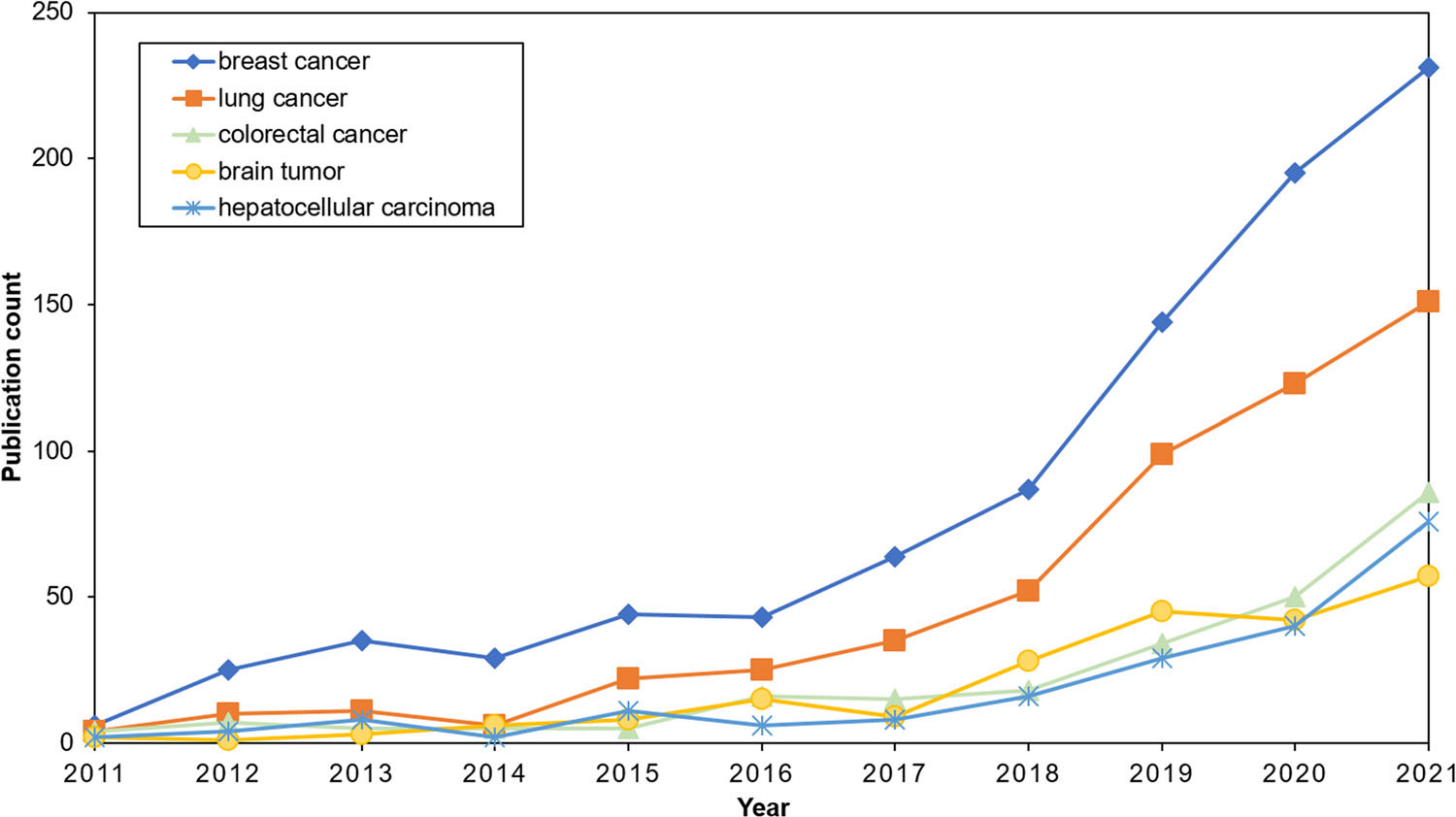
Article trends for the Top 5 types of cancer with the highest *H*‐index values.

Ranked by the *H*‐index, the Top 5 types of cancer research are “breast cancer” (903), “lung cancer” (538), “colorectal cancer” (245), “brain tumor” (216), and “hepatocellular carcinoma” (202). The evolution of the publication numbers of the top *H*‐indexed cancer types is shown in Figure 4. The annual number of article publications on the five cancer types exhibits almost a clear upward trend. *Breast cancer* has a leading position throughout nearly the entire period. *Breast cancer* has experienced a significant increase during the studied period. In particular, *breast cancer* has a dramatic increase during 2018–2021, with 231 articles in 2021. Although the number of *lung cancer* has slightly stagnated or slowly increased during the period 2012–2014, it has exhibited a sharp rise since 2014. As for the other three types of cancer, the number has experienced relatively small increases compared with that of *breast cancer* and *lung cancer*.

### 3.2 Analysis of journal, country/region, institution, and author

The selected 6206 articles are published in a total of 935 different journals. The Top 20 journals ranked by *H*‐index are summarized in Table 2. These journals together account for 32.5% of the articles. This distribution indicates the diversity of journals and disciplines. *Nature Communications* is the most popular journal with the highest *H*‐index value at 36 in the list, followed by *PLoS ONE* (33 *H*‐index) and *Scientific Reports* (21 *H*‐index). Most of the Top 20 journals are directly related to biomedical and computer science, while the others are comprehensive. For example, *Nature Communications* is a multidisciplinary journal dedicated to publishing high‐quality research in all areas of the biological, health, physical, chemical, and Earth sciences [41].

**Table 2:** Top 20 journals ranked by *H*‐index.

Rank	Journal	Country/region	*H*‐index	IF (2020)	Count	Count%
1	*Nature Communications*	United Kingdom	36	13.783	66	1.06
2	*PLoS One*	United States	33	3.041	204	3.29
3	*IEEE Transactions on Medical Imagine*	United States	22	13.936	68	1.10
4	*Scientific Reports*	United Kingdom	21	4.13	351	5.66
5	*BMC Bioinformatics*	United Kingdom	20	3.515	79	1.27
6	*Physics in Medicine and Biology*	United States	19	3.779	92	1.48
7	*Medical Physics*	United Kingdom	18	4.208	133	2.14
8	*Journal of Magnetic Resonance Imagine*	United States	16	4.395	57	0.92
9	*European Radiology*	Germany	14	4.771	126	2.03
10	*Biomed Research International*	United States	12	3.047	65	1.05
11	*IEEE Journal of Biomedical and Health Informatics*	United States	12	6.977	59	0.95
12	*Journal of Biomedical Informatics*	United States	10	7.048	63	1.02
13	*Computer Methods and Programs in Biomedicine*	Netherlands	10	6.271	99	1.60
14	*Computers in Biology and Medicine*	United Kingdom	9	5.591	125	2.01
15	*Artificial Intelligence in Medicine*	Netherlands	8	6.692	60	0.97
16	*IEEE Engineering in Medicine and Biology Magazine*	United States	8	1.09	104	1.68
17	*Journal of Medical System*	United States	7	5.227	67	1.08
18	*IEEE/ACM Transaction Computers Biology Bioinformation*	United States	7	3.413	63	1.02
19	*Computational and Mathematical Methods in Medicine*	Egypt	4	2.426	76	1.22
20	*Studies in Health Technology and Informatics*	Netherlands	3	1.3	60	0.97

From the country/region perspective, a total of 73 countries/regions have participated in the article publications. The United States involves 1851 articles, accounting for 29.83% of the total publications, followed by China (24.96%), the United Kingdom (5.75%), Germany (5.72%), and South Korea (5.16%). From the *H*‐index perspective, the United States also has the highest *H*‐index at 121.4, indicating the high quality of its publications. The other top performers are the United Kingdom, Germany, China, and Canada (Table 3).

**Table 3:** Top 20 countries/regions ranked by *H*‐index.

Rank	Country/region	*H*‐index	Count	Count%	Count rank
1	United States	121.4	1851	29.83	1
2	United Kingdom	59.8	357	5.75	3
3	Germany	54.1	355	5.72	4
4	China*	53.7	1673	26.96	2
5	Canada	51	260	4.19	8
6	France	47.8	196	3.16	11
7	Australia	41.2	162	2.61	12
8	Switzerland	39.2	129	2.08	15
9	Italy	39.1	248	4.00	9
10	Netherlands	38.6	241	3.88	10
11	Spain	38	148	2.38	14
12	Hong Kong, China	37.4	78	1.26	18
13	Israel	35.1	94	1.51	17
14	Singapore	34.5	58	0.93	19
15	Japan	32.5	271	4.37	7
16	South Korea	31.5	320	5.16	5
17	Belgium	31.2	54	0.87	20
18	Taiwan, China	30.6	161	2.59	13
19	India	30.1	315	5.08	6
20	Sweden	29.8	97	1.56	16

* The data is for the mainland China, and does not include Hong Kong, Macao and Taiwan.

The level of scientific research, scientific and technological strength, and the distribution of scientific research achievements can be revealed by the analysis of research institutions [42]. Table 4 depicts the Top 20 institutions ranked by *H*‐index. Among them, 15 are from the United States. Harvard University (323 publications and 98.9 *H*‐index) and Stanford University (304 publications and 98.05 *H*‐index) ranked in the Top 2. Taking into consideration of all listed index metrics, Harvard University has the top‐rank position, reflecting the high quality of its articles as well as its dominant position in the field. Other influential institutions include the University of Toronto (*H*‐index as 94.15). Other productive institutions include the Massachusetts Institute of Technology (204 articles).

**Table 4:** Top 20 institutions ranked by *H*‐index.

Rank	Institution	Country/region	*H*‐index	Count	Count%
1	Harvard University	United States	98.9	323	5.20
2	Stanford University	United States	98.05	304	4.90
3	University of Toronto	Canada	94.15	181	2.92
4	Massachusetts Institute of Technology	United States	93.7	204	3.29
5	University of Washington	United States	92.85	122	1.97
6	University of Pennsylvania	United States	91.6	133	2.14
7	Johns Hopkins University	United States	49.6	117	1.89
8	Chinese Academy of Sciences	China	48	139	2.24
9	University of California, San Francisco	United States	47.8	68	1.10
10	University of California, Los Angeles	United States	47.8	56	0.90
11	Columbia University	United States	47.5	105	1.69
12	Duke University	United States	47.3	112	1.80
13	University of Michigan	United States	46	112	1.80
14	Yale University	United Kingdom	45.7	48	0.77
15	University of Oxford	United Kingdom	43	82	1.32
16	Tsinghua University	China	42.15	48	0.77
17	University of Tokyo	Japan	41	75	1.21
18	Northwestern University	United States	40.5	62	1.00
19	Georgia Tech	United States	40.35	45	0.73
20	Fudan University	China	35	102	1.64

A total of 12,355 authors participate in the publication of the 6206 articles. 77.69% of the authors have only one article. Table 5 shows the main statistical characteristics of the Top 20 authors ranked by *H*‐index, among which 8 are from the United States. *Jing Zhang* from China is in first place with the highest *H*‐index at 47, and the number of his articles ranks fourth. Next in importance include *Yang Zhang* from the United States, *Di Dong* from China, and *Jeonghoon Lee* from Korea. *Yang Lei* from the United States, *Renato Cuocolo* from Italy, and *Lin Lu* from the United States are the Top 3 in terms of article count.

**Table 5:** Top 20 authors ranked by *H*‐index.

Author	Country/region	*H*‐index	Count	Author	Country/region	*H*‐index	Count
Jing Zhang	China	47	8	Renato Cuocolo	Italy	19	13
Yang Zhang	United States	44	7	Hidetaka Arimura	Japan	19	7
Di Dong	China	34	7	Ashirbani Saha	United States	18	7
Jeonghoon Lee	Korea	30	5	Isaac Shiri	Switzerland	18	5
Yang Lei	United States	27	18	Gilmer Valdes	United States	18	5
Hao Chen	Hong Kong, China	27	7	Lin Lu	United States	17	9
Lei Zhang	China	26	7	Dan Nguyen	United States	16	8
Tonghe Wang	United States	24	5	Arnaldo Stanzione	Italy	16	6
Aditya V Karhade	United States	21	8	Huiyan Jiang	China	15	8
Yoshiko Ariji	Japan	21	5	Michele Avanzo	Italy	15	5

**Figure 5:**
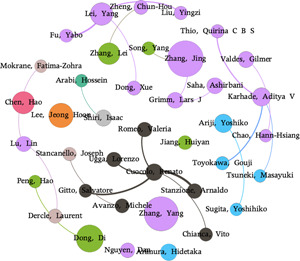
The collaboration network of the top authors ranked by *H*‐index.

With the help of Gephi, a co‐occurrence analysis of coauthor relations of the 20 authors (listed in Table 5) is generated in the form of a social network with 39 nodes and 22 links, as shown in Figure 5. The node size donates the article count for each author, while the node color represents the country/region of the authors. For example, the purple color denotes the United States. *Renato Cuocolo* and *Arnaldo Stanzione* have collaborated the most (4 articles), followed by *Yang Lei* and *Yabo Fu* (3) as well as *Renato Cuocolo* and *Salvatore Gitto* (3).

### 3.3 The evolution of research topics

The LDA model is applied to overall thematic detection from the article publications. Model perplexity is a measure of how well a probability distribution or probabilistic model predicts sample data. In brief, a lower perplexity value indicates a better topic model on the data. Thus, we use different numbers of topics to compute the model perplexity and identify the best model with 16 topics along with representative terms, topic proportions, and suggested topic labels, as reported in Table 6. The Top 5 topics are within the research field including “Image processing” (15%), “Patient analysis” (11.8%), “Test and evaluation” (10.7%), “Gene analysis” (8.4%), and “Radiomics analysis” (7.9%). Most topics identified are recognizable compared with existing research reviews in the research field. To further understand the topics, some representative topics are interpreted hereby. Topic 2 contains words such as “patient,” “cancer,” “risk,” “survival,” “clinical,” “prediction,” “treatment,” and “prognostic,” thus it pertains to patient information analysis. For machine learning to improve cancer research, the research is closely related to patient diagnostic information. Topic 3 discusses the method of testing and training, including terms such as “accuracy,” “test,” “validation,” “specificity,” and “compared.” Most relevant studies divide data into training sets and test sets so as to develop and test the performance of developed algorithms in the field.

**Table 6:** Top 16 most frequent terms for the 16 detected topics.

Rank	Topic label	Top high‐frequency terms	Count%
1	Image processing	Image, proposed, feature, training, information, approach, tumor, accuracy, data set, accuracy	15
2	Patient analysis	Patient, cancer, risk, survival, clinical, prediction, machine learning, treatment, prognostic, stage	11.8
3	Test and evaluation	Accuracy, performance, test, validation, specificity, diagnostic, AUC, lesions, compared, algorithm	10.7
4	Gene analysis	Gene, expression, cancer, analysis, identified, biomarker, related, cell, network, sample	8.4
5	Radiomics analysis	Feature, radiomics, image, patient, CT, MRI, AUC, performance, validation, machine learning	7.9
6	Classification	Cancer, prediction, performance, proposed, classification, selection, algorithm, feature, data set, machine learning	6.9
7	Machine learning algorithms	Feature, SVM, classification, accuracy, classifier, texture, analysis, random, selection, forest	6.5
8	Deep learning methods	Image, breast, deep learning, neural, network, segmentation, classification, cancer, CNN, convolutional	5.7
9	Medical informatics	Cancer, clinical, machine learning, AI, research, medical, health, diagnosis, review, information	5.2
10	Prostate cancer	Prostate, dose, CT, segmentation, radiotherapy, radiation, image, volume, deep learning, mean, planning	4.7
11	Cell	Cell, image, carcinoma, cancer, deep learning, tumor, melanoma, diagnosis, slide, pathologists	4.2
12	Breast cancer	Cancer, tumor, breast, molecular, mutation, status, methylation, mutations, subtypes, genome, DNA	3.2
13	Brain cancer	Tumor, brain, liver, HCC, image, grade, glioma, patient, contrast, gliomas, hepatocellular	3.1
14	Drug treatment	Cell, drug, cancer, machine learning, response, omics, blood, multiple, prediction, density	2.7
15	Lung cancer	Lung, CT, cancer, deep learning, nodules, metastasis, node, detection, pulmonary, thyroid	2.2
16	Raman	Cancer, proteins, Raman, spectroscopy, serum, acid, controls, compounds, inhibitors, human	1.8

Abbreviations: ACC, adrenocortical carcinoma; AI, artificial Intelligence; CNN, convolutional neural network; CT, computed tomography; HCC, hepatocellular carcinoma; MRI, magnetic resonance imaging; SVM, support vector machine.

**Figure 6:**
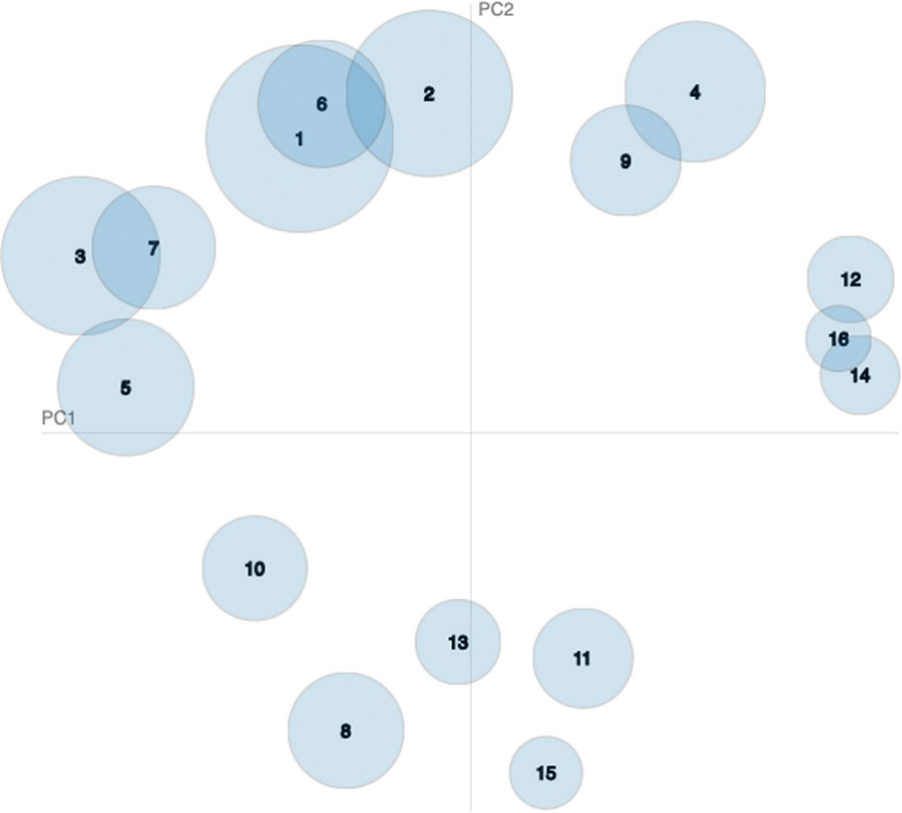
The analysis of topic distributions and their overlaps using multidimensional scaling.

To analyze the topic distributions and their overlap, an intertopic distance map via multidimensional scaling is generated, as shown in Figure 6. PC1 and PC2 represent principal component 1 and principal component 2, respectively. The overlapping graphs represent the two topics addressed more frequently in the same article. For example, there are multiple overlapping parts of “image technology” and “classifier,” indicating that there are also a large proportion of “classifiers” in the articles with a high proportion of “image technology.”

By utilizing a K‐means clustering model, article keywords are grouped into five clusters, including “Machine Learning,” “Imaging diagnostic,” “Computer‐assisted Radiotherapy,” “Cancer,” and “Patient demographics,” as shown in Table 7. Comparing the results with that of topic modeling, most of the identified groups by K‐means clustering are more concise and interpretable. This may be caused by using only article keywords, KeyWords Plus, and PubMed MeSH for achieving high‐quality keywords, while topic modeling utilizes additional automatically extracted keywords from the title and abstract.

**Table 7:** The result of K‐means clustering.

Cluster	Cluster label	Keyword
1	Machine learning	Machine learning, support vector machine, classification, analysis
2	Imaging diagnostic	Imaging, diagnostic, pathology, retrospective studies, tomography
3	Computer‐assisted radiotherapy	Computer‐assisted, modeling, radiotherapy, image processing
4	Cancer	Genetics, neoplasms, tumor, cell, method
5	Patient demographics	Aged, middle‐aged, adult, male, female, humans

**Figure 7:**
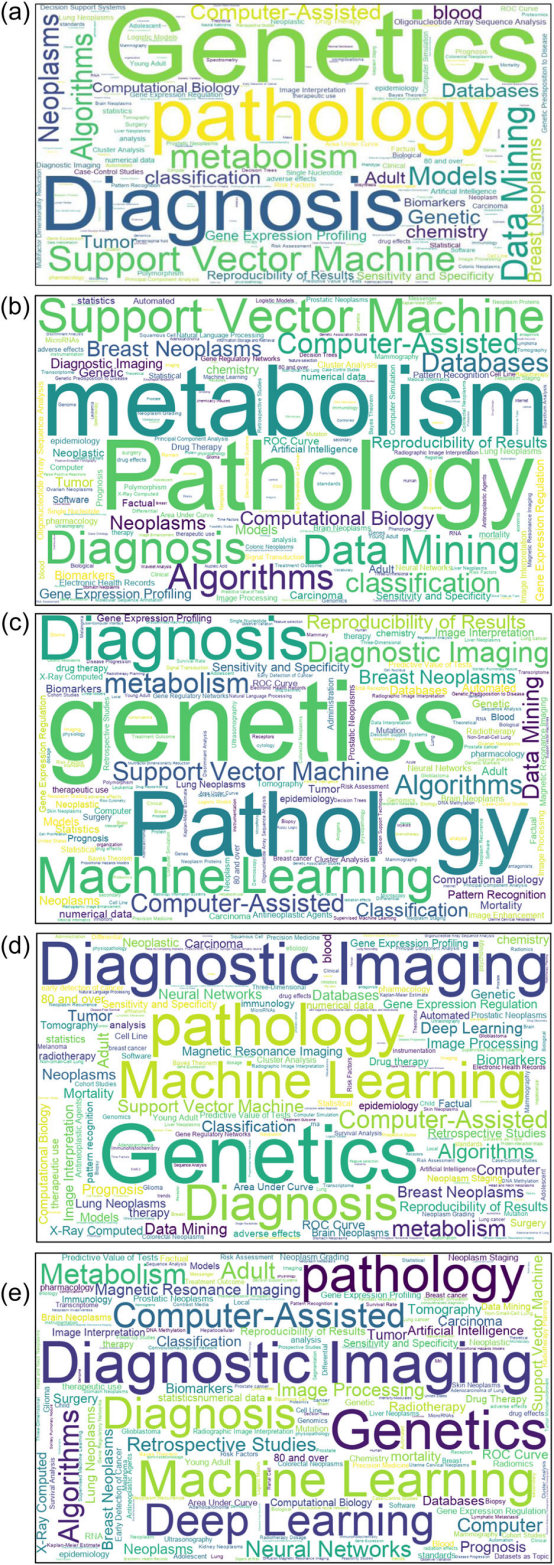
The evolution of keyword clouds of during the five time periods (a) 2011–2012, (b) 2013–2014, (c) 2015–2016, (d) 2017–2018, and (e) 2019–2020.

We further explore topics evolution in terms of frequency every 2 years on the basis of five time periods (2011–2012, 2013–2014, 2015–2016, 2017–2018, and 2019–2020). For each period, keywords are visualized as a word cloud, as shown in Figure 7, with font size indicating word frequency. From the results, the research topics have become more diverse with time while “Genetics,” “Computer‐Assisted,” “Support Vector Machine,” and “classification” are always the core focuses of the field. Some keywords draw more and more attention, for example, “Diagnostic Imaging,” “Machine Learning,” “Deep Learning,” and “Neural Networks” started to appear in the period 2015–2016 and have gained continuing attention since then.

## 4 DISCUSSION

In the early 21st century, research with the application of machine learning methods has been already available. Gradually, with the development of machine learning, more and more researchers conduct cancer studies utilizing machine learning technologies. Our research topic analysis using topic modeling illustrates how machine learning‐driven cancer research has evolved during the past 10 years. On the whole, the research topics have become more diverse with time. Undoubtedly, keywords such as “support vector machine,” “classification,” and “deep learning” have been frequently used during the study period, resulting in the fact that machine learning is the focus of this study and the application of classification is also widely used in the diagnosis of diseases as well as medical data analysis. In limited research work, classification is used as one of the major data analysis strategies. As time went by, machine learning‐related vocabulary is becoming more and more abundant, which indicates a growing interest in applying machine learning in different directions of cancer research.

During the period 2011–2021, “genetics” occurs the most (1763 occurrences), indicating that machine learning technologies have been widely used in cancer research concerning genetics. In the studies of cancer, “breast cancer,” “lung cancer,” and “colorectal cancer” are the hot research focuses. With the change of time, more and more types of cancer are being studied. That was to say, research topics related to cancer that used machine learning technologies have become more diverse with time. In these studies, machine learning mainly serves as an auxiliary tool. The new keyword “diagnosis” has subsequently joined in since the year 2011 and has been always as frequent in publications, which show that AI technologies are popular in the diagnosis of cancer diseases.

As for research tools and methods, distinct changes include imaging tools like “radiographic image,” “magnetic resonance imaging,” and “diagnostic imaging,” as well as machine learning technologies like “support vector machines” and “convolutional neural networks.” The study of radiographic images started earlier, and “magnetic resonance imaging” has consistently been the research focus during the past decade. Since 2015, the frequencies of “diagnostic imaging” have also increased significantly, from ranking fifth to first, indicating that more diagnostic imaging‐related studies started to utilize machine learning technologies.

The keyword “support vector machines” has been among the top research hotspots since 2011, serving as the most frequently applied algorithm, and the keywords “classification” and “test‐validation” are also keywords of high frequency. The ranking position of the keywords “machine learning” has continued to increase over time going on. This indicates that there is an increasing interest among scholars in utilizing machine learning methods for cancer studies. The keyword “deep learning” appeared in the period 2015–2016 and rose to rank fifth in the 2017–2018 period. This indicates a dramatic fast increasing interest among scholars in applying deep learning technologies to cancer studies. We can deduce that the incorporation of deep learning technologies into cancer research is probably still a prominent trend in the near future. The keywords “image” and “segmentation” mainly refer to the data analysis of medical images, for example, automatic identification and segmentation of different brain regions in MRI images, and the segmentation of gray matter and white matter. The segmentation effect varied a lot depending on the use of different algorithms. Thus, it is necessary to continuously develop and improve the relevant algorithms to obtain better image segmentation.

Other highly frequently used keywords included “model,” “algorithm,” “feature,” “pattern,” and “performance.” These keywords are related to the research on the improvement of statistical models and algorithms.

There is no doubt that machine learning currently has undisputed potential, which may influence the field of cancer research deeply, the overall development of precise examination, diagnosis, and treatment of cancer diseases seems to be promising. Future challenges in applying machine learning to cancer research may mainly lie in personalized data collection, data normalization, aiding diagnostic confidence decisions, and so on. The findings of this study complement existing subjective and evaluative publication reviews on worldwide cancer research using machine learning methods. In addition, exploring the emerging research trends of machine learning in cancer research can promote the development of clinical research. More importantly, understanding the contributions of the most productive scholars and their research helps other researchers to follow and collaborate. Certainly, this study still has some limitations. First, data were retrieved only from one source, PubMed. More data sources such as Embase and Scopus could be involved to enhance publication coverage. Second, the search terms might not be sufficient enough to retrieve all relevant publications in the study field.

## 5 CONCLUSION

By retrieving and studying 6206 articles from PubMed about cancer research using machine learning methods during 2011–2021, this paper applies bibliometric and visualized methods to uncover a continuingly growing interest in this research field. Continuing development of the research is indicated by the trend analysis of the articles. This paper also recognizes top journals, influential countries/institutions, as well as authors, scientific collaborative relations among authors, and reveals distributions and evolutions of topics. First, understanding the contributions of the most productive scholars and their research helps other researchers to grasp future research interests and to guide future research routines. In addition, interdisciplinary collaborations can be revealed and promoted. The potential use of machine learning in various aspects of cancer therapy and its powerful computational power has facilitated its utilization in medical research. At the same time, the application of machine learning in cancer research is also conducive to the research breakthrough of machine learning.

## AUTHOR CONTRIBUTIONS

**Lianghong Lin**: Formal analysis (equal); methodology (equal); validation (equal); visualization (equal); writing—original draft (equal). **Likeng Liang**: Validation (equal); writing—review and editing (equal). **Maojie Wang**: Investigation (equal); writing—review and editing (equal). **Runyue Huang**: Conceptualization (equal); writing—review and editing. **Mengchun Gong**: Investigation (equal); writing—review and editing (equal). **Guangjun Song**: Resources (equal); software (equal). **Tianyong Hao**: Funding acquisition (equal); project administration (equal); supervision (equal); writing—review and editing (equal).

## CONFLICT OF INTEREST STATEMENT

Professor Mengchun Gong and Tianyong Hao are members of the *Cancer Innovation* Editorial Board. To minimize bias, they were excluded from all editorial decision‐making related to the acceptance of this article for publication. The remaining authors declare no conflict of interest.

## ETHICS STATEMENT

Not applicable.

## INFORMED CONSENT

Not applicable.

## Data Availability

The data used in the paper is publicly available.
